# The Intersection between Ocular and Manual Motor Control: Eye–Hand Coordination in Acquired Brain Injury

**DOI:** 10.3389/fneur.2017.00227

**Published:** 2017-06-01

**Authors:** John-Ross Rizzo, Maryam Hosseini, Eric A. Wong, Wayne E. Mackey, James K. Fung, Edmond Ahdoot, Janet C. Rucker, Preeti Raghavan, Michael S. Landy, Todd E. Hudson

**Affiliations:** ^1^Department of Rehabilitation Medicine, New York University Langone Medical Center, New York, NY, United States; ^2^Department of Neurology, New York University Langone Medical Center, New York, NY, United States; ^3^Department of Psychology and Center for Neural Science, New York University, New York, NY, United States; ^4^Department of Ophthalmology, New York University Langone Medical Center, New York, NY, United States

**Keywords:** coordination, eye, hand, stroke, brain injuries

## Abstract

Acute and chronic disease processes that lead to cerebral injury can often be clinically challenging diagnostically, prognostically, and therapeutically. Neurodegenerative processes are one such elusive diagnostic group, given their often diffuse and indolent nature, creating difficulties in pinpointing specific structural abnormalities that relate to functional limitations. A number of studies in recent years have focused on eye–hand coordination (EHC) in the setting of acquired brain injury (ABI), highlighting the important set of interconnected functions of the eye and hand and their relevance in neurological conditions. These experiments, which have concentrated on focal lesion-based models, have significantly improved our understanding of neurophysiology and underscored the sensitivity of biomarkers in acute and chronic neurological disease processes, especially when such biomarkers are combined synergistically. To better understand EHC and its connection with ABI, there is a need to clarify its definition and to delineate its neuroanatomical and computational underpinnings. Successful EHC relies on the complex feedback- and prediction-mediated relationship between the visual, ocular motor, and manual motor systems and takes advantage of finely orchestrated synergies between these systems in both the spatial and temporal domains. Interactions of this type are representative of functional sensorimotor control, and their disruption constitutes one of the most frequent deficits secondary to brain injury. The present review describes the visually mediated planning and control of eye movements, hand movements, and their coordination, with a particular focus on deficits that occur following neurovascular, neurotraumatic, and neurodegenerative conditions. Following this review, we also discuss potential future research directions, highlighting objective EHC as a sensitive biomarker complement within acute and chronic neurological disease processes.

## Introduction

Acute and chronic disease processes that lead to cerebral injury can often be clinically challenging diagnostically, prognostically, and therapeutically. Neurodegenerative processes are one such elusive diagnostic group, given their often diffuse and indolent nature, creating difficulties in pinpointing specific structural abnormalities that relate to functional limitations. Historically, experiments have concentrated on cerebral lesion-based approaches, significantly improving our understanding of the neurophysiology and underscoring the sensitivity of behavioral biomarkers to detect as well as predict the outcomes of cerebral injury. These focal lesion-based models and associated biomarkers can be combined synergistically and have significant potential in shedding light on acute and chronic neurological disease processes.

Eye–hand coordination (EHC) can be defined as the complex relationship between our visual system and our manual motor system. Visually guided reaching, grasping, and object manipulation depend on the ability to visually decipher environmental details and finely coordinate motor responses of the eye and hand to produce controlled, rapid and accurate movements. Independent deficits of either ocular or manual motor control have been studied extensively after acquired brain injury (ABI). More recently, the coordination between eye and hand movements in patients with central nervous system injury, as related to neurovascular, neurotraumatic, and neurodegenerative conditions, has been highlighted as a critical concept in understanding brain-behavior relationships.

Over the course of the past two decades, a number of studies have demonstrated that EHC deficits (i.e., eye–hand incoordination or dyssynergia) resulting from ABI are important thematic concepts within the field of rehabilitation following neurological injury (1–3). In response, a focused review was performed on the PubMed database using a series of key words that included the following phrases and/or words: eye–hand coordination, acquired brain injury, stroke, cerebrovascular accident (CVA), traumatic brain injury, and brain injury (including acute, subacute, and chronic time scales). The research included articles published over the past two decades. A total of 74 articles were surveyed, which varied significantly in scope and merit.

The aim of this narrative review on EHC was to clarify its conceptual importance in the setting of ABI, to improve understanding neuroanatomically, and to address implications therapeutically. The articles reviewed were focused on EHC or the integration of visual input secondary to ocular motor control and manual motor output and related pathology, including neurovascular, neurotraumatic, and neurodegenerative conditions. The overarching goal of this review is to engender dialogue between clinicians and scientists in a framework that will provide clarity, improve comprehension and precipitate translational, clinical research.

## Literature Search Strategy

**Figure 1:**
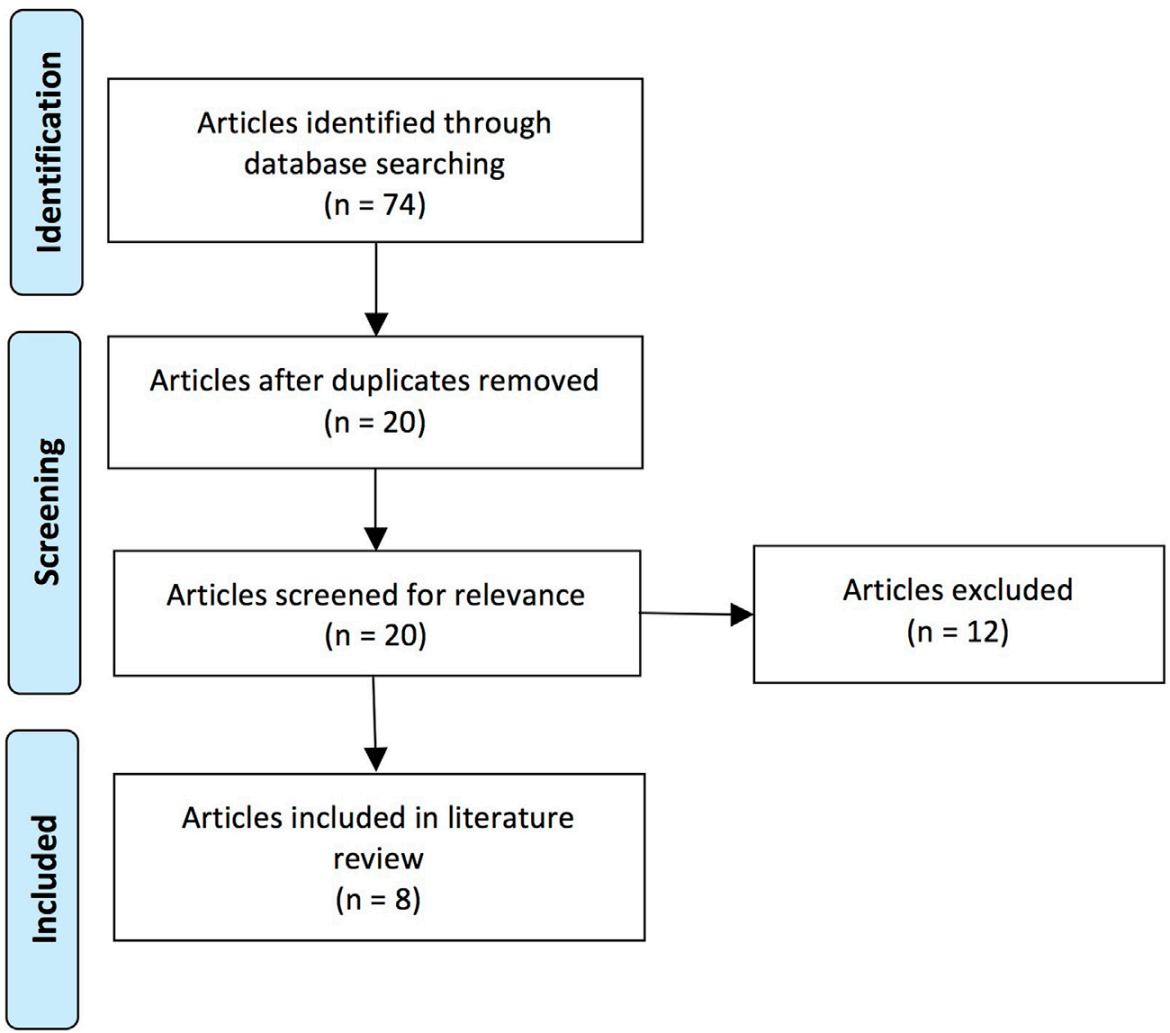
**Flow diagram of literature search**.

Our literature review was performed by J.R. and E.W. on publications available in the National Center for Biotechnology Information’s PubMed database using key words containing the phrase “eye hand coordination” and key words relevant to ABI (specific key words are listed in Table 1). The search of the literature included seminal and contemporary peer-reviewed articles on EHC in the setting of ABI, including injuries that were either secondary to trauma or CVAs. The research articles spanned publication dates between 1998 and 2015. The quality and the relevance of the resultant literature varied significantly in caliber and applicability. Articles were utilized based on their pertinence to ABI and its associated effects on EHC. Pertinence was determined by consensus between two authors based on whether there was a thematic focus on EHC, and also discussion of at least one of the patient populations of interest. A total of 74 articles were originally reviewed (surveyed); this compilation was ultimately distilled to 8 pertinent (utilized) references (see Figure 1 and Table 2).

**Table 1:** **Literature search strategy details**.

Key words	Articles surveyed	Articles utilized
Eye hand coordination acute brain injury	3	2
Eye hand coordination chronic brain injury	2	2
Eye hand coordination subacute brain injury	0	0
Eye hand coordination ABI	1	1
Eye hand coordination stroke	14	5
Eye hand coordination acute stroke	14	5
Eye hand coordination chronic stroke	0	0
Eye hand coordination CVA	14	5
Eye hand coordination cerebrovascular accident	14	5
Eye hand coordination traumatic brain injury	5	3
Eye hand coordination TBI	1	1
Eye hand coordination traumatic injury	6	3
Total listed items	74	32
Total articles (duplicate articles removed)	20	8

**Table 2:** **Key comparisons of the articles utilized in the review following the literature search and selection process**.

Author	Year	Journal	Cohort	Obj. eye^a^	Obj. hand^b^
Caeyenberghs et al. (1)	2009	*J Head Trauma Rehabil*	ABI	[−]	(+)/2D
Caeyenberghs et al. (210)	2010	*Neuropsychologia*	TBI	[−]	(+)/2D
Brown et al. (215)	2015	*BMC Sports Sci Med Rehabil*	TBI	[−]	(+)/2D
Gao et al. (2)	2010	*J Rehabil Med*	CVA	[−]	(+)/2D
Ghika et al. (3)	1998	*Clin Neurol Neurosurg*	CVA	[−]	(−)
Tsang et al. (142)	2013	*Am J Phys Med Rehabil*	CVA	[−]	(+)/2D
Procacci et al. (106)	2009	*Neurocase*	CVA	[+]	(+)/2D
Vesia et al. (74)	2012	*Exp Brain Res*	Review	[n/a]	(n/a)

*^a^Obj. eye = objective eye recording was performed [+] or not performed [−]*.

*^b^Obj. hand = objective hand recording was performed (+) or not performed (−) and, if performed, were the recordings in 1D, 2D, or 3D*.

*ABI = acquired brain injury; TBI = traumatic brain injury; CVA = cerebrovascular stroke*.

## EHC Definition

Eye–hand coordination is the complex relationship between the visual and manual motor systems, at the intersection between vision and dexterity. EHC depends on vision to aid in directing goal-oriented hand movements, including pointing, reaching, grasping, object manipulation, and tool use, and encompasses many functionally relevant motor activities (4, 5). Optimal coordination relies on precise ocular motor control for high acuity visual perception and sound manual motor control, yielding robust effector coaction (6, 7). This visuomotor integration requires complex motor programs and near continuous, multimodal sensory feedback, and predictions thereof, to produce controlled and rapid task-specific movements (8).

## EHC Neurophysiology and Neuroanatomy

### The Visual System (Eye)

Primary visual cortex (V1), also known as striate cortex, is the first cortical region that processes visual input. V1 is located in the posterior pole of the occipital lobe. It mainly serves to process primitive visual features, such as bars of a specific orientation or edges and contours of solid objects within a specific portion of the retina’s visual field. From V1, visual processing continues through a sequence of adjacent cortical regions known as extrastriate cortex. A fundamental organizing principle of these visual areas is a topographic representation of the contralateral visual field. The spatial layout of a scene is represented in an orderly manner across a population of neurons that reflect input from the retina. This population of neurons constitutes a visual field map whereby adjacent neurons represent adjacent points in space (9), preserving the spatial layout of the retinal image in each of these cortical areas. This systematic organization is computationally and metabolically efficient as it shortens connection lengths between similarly tuned neurons. Interestingly, topographic organization extends beyond retinotopic coordinate space. Relevant for EHC, other areas represent space in head-centered coordinates (10–12), or a combination of coordinate systems (13, 14). The interactions between these areas likely facilitate sensorimotor transformations fundamental to EHC. Extrastriate regions (such as V2/V3), which emanate rostrally from V1, are believed to be responsible for processing features of progressively increasing complexity (15–17). This processing stream bifurcates into a ventral “what” pathway, processing object identity and visual features, and a dorsal “where” pathway, processing spatial attention and movement (15, 18). The dorsal pathway has also been implicated in processing visual input for predictive and anticipatory movements, including those coordinated between the eye and hand (17, 19, 20). The dorsal and ventral streams are thought to aid EHC (21, 22).

### The Ocular Motor System

**Figure 2:**
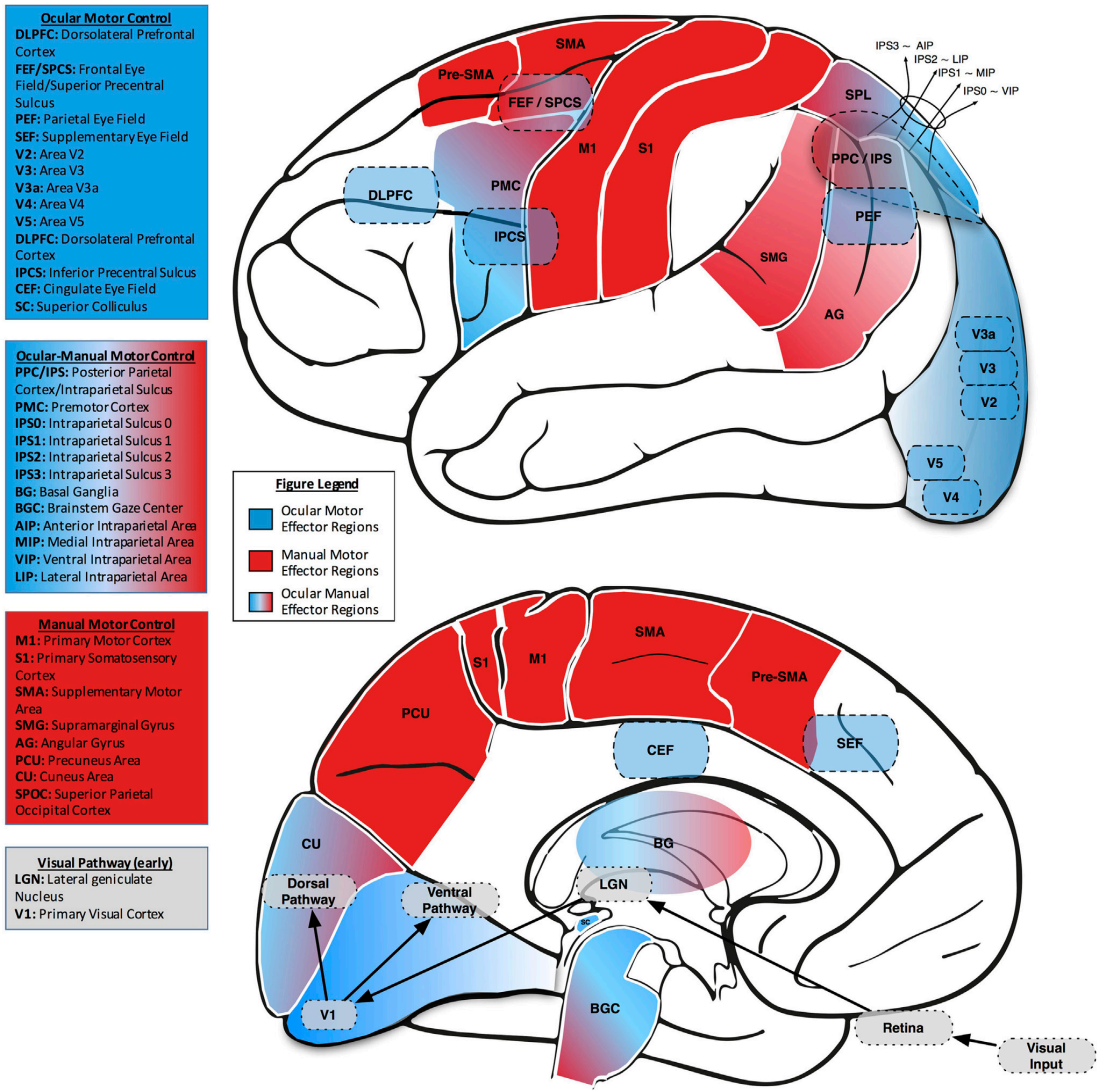
**Lateral (upper) and midsaggital (lower) views of the human brain, labeled with neuroanatomical regions of interest related to eye–hand coordination**. In both views, ocular motor areas are colored blue, manual motor areas red, and combined ocular-manual motor areas a blend of red and blue.

In order to examine our environment, we alternate between fixating a point of interest and making fast, darting eye movements (saccades) from one point of interest to another. For well over a century, scientists have measured saccades to investigate the link between brain and behavior (23, 24). Broadly, along with the subcortical superior colliculus (SC), four cortical areas contribute to the control of saccades: the frontal eye field (FEF), the supplementary eye field (SEF), the parietal eye field (PEF), and the cingulate eye field (CEF). Each region appears to play a distinct role in controlling eye movements. The FEF, SEF, and PEF directly project to the SC, while the CEF influences ocular motor control more indirectly through connections with the FEF, SEF, and PEF (25–29). Additionally, the FEF connects directly to the brainstem ocular motor nuclei, which house the ocular motor neurons that innervate the extraocular muscles (Figure 2).

#### Frontal Eye Field

The FEF is crucial for the preparation and execution of voluntary saccades to either external (visually guided saccades) or internal targets (memory-guided saccades) (30–34). The majority of research characterizing the FEF has been with respect to the monkey ocular motor system, ever since Ferrier discovered that electrical stimulation of the FEF elicited eye movements (23). In the monkey, FEF is located in the anterior bank of the arcuate sulcus, just posterior to the principal sulcus (31). The FEF both projects to and receives connections from numerous cortical and subcortical brain regions (35, 36). It is retinotopically organized and primarily comprised of neurons that contribute to the holding or shifting of gaze (fixation neurons and saccade neurons, respectively) or neurons that generally respond to stimuli within their receptive field (visual neurons) (37, 38). Neurons within the FEF also have a preference for the contralateral visual field (31). In monkeys, there is also a rough topographic organization with regards to saccade amplitude. The superior portion of the FEF is responsible for generating larger amplitude saccades and shares connections with the dorsal visual stream, while the inferior portion of the FEF is responsible for generating smaller amplitude saccades and shares connections with the ventral visual stream (39). Interestingly, this topography and connectivity organized by dorsal and ventral streams has yet to be demonstrated in the human FEF. Instead, the putative human homolog of FEF, located in the superior precentral sulcus (SPCS), is organized into distinct visual field map clusters similar to early visual cortex (40).

#### Supplementary Eye Field

The SEF is involved in more indirect aspects of saccade control, such as monitoring the consequences or context of eye movements (41) and coordinating sequences of successive saccades (42, 43). Although believed to typically be found in the posteromedial part of the human superior frontal gyrus, there exists a great amount of variability between individuals in the exact location of the SEF, thus rendering it difficult to define by anatomy alone (44). The activity of neurons in the SEF is modulated by target position in multiple reference frames, aiding in maintaining eye position despite changes in body and head position (14, 45). This region receives both sensory and motor inputs and supplies outgoing connections to both the FEF and PEF (46). Although electrical stimulation of an FEF neuron elicits an eye movement of a fixed magnitude and direction, SEF stimulation elicits an eye movement to a fixed region of the visual field relative to the position of the head, irrespective of the starting position of the eye (47). Although much less is known about topographic organization in SEF, a recent human neuroimaging study suggests it also contains an orderly map of continuous space similar to other visual areas (48).

#### Parietal Eye Field

Visual input is received by the PEF and aids in triggering reflexive saccades toward visual stimuli found within the peripheral field of vision, as well as managing alterations in attention (49) and performing memory-guided saccades (40, 50). The PEF is located in the lateral intraparietal (LIP) area in monkeys and the intraparietal sulcus (IPS) in humans, and contains strong and reciprocal connections with the FEF. Similar to early visual areas, the IPS contains multiple visual field maps of contralateral space that have led to further parcelation (34, 51). These subregions are labeled numerically (IPS0, IPS1, IPS2, IPS3, etc.), starting from the most posterior area, IPS0, which borders V3A/V3B. Each of these subdivisions are activated during human neuroimaging studies involving eye movements (52). Therefore, which of these individual maps, if any, directly correspond to subdivisions of the monkey IPS (LIP, AIP, VIP, MIP) is still up for debate (52–55). It is also possible that some of the retinotopic IPS subdivisions are unique to humans.

#### Cingulate Eye Field

The CEF is involved in intentional but not reflexive saccade control (56), and projects to both the FEF and SEF (57). In non-human primates, the CEF is located on the medial wall in each hemisphere, ventral and partly anterior to the SEF. In humans, however, the CEF is located more posterior and ventral to the SEF (57). In humans, lesions of the CEF impair many types of saccades, including sequences of visually-guided saccades, memory-guided saccades, and antisaccades (56). Compared to the other ocular motor regions listed here, the CEF is the least studied and least understood.

#### Superior Colliculus

The SC plays a crucial role in saccade execution, as it projects directly to the brainstem ocular motor nuclei. It receives projections from a multitude of areas including FEF, SEF, and PEF. Like FEF, electrical stimulation of SC elicits saccades of a particular magnitude and direction. SC is also organized similarly to FEF, except in a rostral-caudal, rather than an inferior–superior, gradient of increasing saccade amplitude. Recent human neuroimaging studies have demonstrated that human SC contains a retinotopic map of the entire contralateral visual field (58, 59). In non-human primates, lesions of the SC alone impair but do not abolish eye movements, but lesions of SC and FEF together have catastrophic consequences for eye movements that do not recover with time (60).

#### Other Areas

The aforementioned areas are clearly not an exhaustive list of brain regions associated with ocular motor control, although they are the most studied. For example, dorsomedial frontal cortex, sometimes referred to as the presupplementary motor area, is critical for inhibition of reflexive saccades in humans (61). It has also been implicated in selecting among competing movements during action selection (62). Additionally, V1 also plays a role in ocular motor control and has projections directly to the SC. In the rhesus monkey, electrical stimulation of V1 can elicit saccades, but the required level of stimulation is much higher than what is necessary to elicit saccades via FEF or SC stimulation (63).

The role of human dorsolateral prefrontal cortex (dlPFC) in ocular motor control is still unclear. Electrophysiological and lesion studies in non-human primates show that the dlPFC contains spatially selective neurons that are critical for memory-guided saccades (64, 65). However, lesions to human dlPFC do not impair memory-guided saccades (34), and do not show spatial selectivity (34, 66). A handful of studies have examined the effects of transcranial magnetic stimulation (TMS) on dlPFC during a memory-guided saccade task (67–69). These results seem to parallel the results from non-human primate lesion studies, finding effects of TMS on memory-guided saccade performance. However, it is likely that the stimulation site in these studies overlapped with the FEF. All three papers used the identical method to localize and define dlPFC, first finding the motor hand area and then moving anteriorly a few centimeters; this method has also been described as an effective way to localize the human FEF (70). A more recent study using TMS to disrupt activity in human dlPFC found no impairment on memory-guided saccades (48).

### The Manual Motor System (Hand)

Within EHC, the end goal is to place the hand/finger(s) or the manual effector in the position required for motor program execution or, in a dynamic sense, to work seamlessly and reciprocally with the eye to build and actualize complex motor programs. The neuroanatomical reach network most directly responsible for voluntary movements of the arm and hand includes motor cortical regions such as primary motor cortex (M1) and the supplementary and premotor cortices. The primary motor cortex begins on the anterior wall of the central sulcus and continues rostrally to comprise what is the anterior paracentral lobule. It is the cortical region responsible for the collective generation of action potentials that relay neural information to the descending corticospinal tract to produce hand movements (71). The premotor cortex (PMC) is located anterior to the primary motor cortex (M1) and in a lateral position from midline; this region is in close spatial proximity to the inferior precentral sulcus (70). PMC is the planning region for anticipatory movements, provides spatial guidance during hand movements, and processes the sensory input used to aid the guidance of hand movements. The supplementary motor cortex is closer to the midline and anterior to the primary motor cortex, and is used to plan sequential manual movements. These motor areas supply the bulk of the neurons whose axons compose the corticospinal tract (in conjunction with smaller inputs from somatosensory, posterior parietal, and cingulate cortex), which travels through the internal capsule and pons, decussates at the level of the medulla, and ultimately activates the alpha motorneurons in the spinal cord (primarily the lower cervical and first thoracic levels) either directly or via spinal interneurons.

This cortical reach network is supplemented by a larger network of cortical and subcortical regions, including the posterior parietal cortex (PPC), somatosensory cortex, basal ganglia, and cerebellum. The PPC is an associative region that translates visual information and input from the somatosensory cortex into motor commands (72, 73). Based on functional neuroimaging, TMS studies, and human case series with parietal injuries, a functional topography for reach, as it relates to the planning and control of visuomotor action, has been described within the human PPC (74). More specifically, midposterior intraparietal sulcus (mIPS), superior parietal occipital cortex (SPOC), and angular gyrus (AG) are reach-specific areas (Figure 2). Three main aspects in reach-dominant areas include effector specificity, hemispheric laterality and computational specificity. The area posteromedial to IPS contributes to the planning of reaching, while the area anterolateral to the IPS has a role in grasp-related information integration. Cortex anterior to the intraparietal area (AIP) is involved in object-directed hand grasping and hand preshaping. In hemispheric lateralization, bilateral activation due to reaching with more emphasis on contralateral movements has been identified (75).

The anterior portion of IPS monitors the compatibility of a planned reach/grasp with outgoing movement commands and incoming sensory inputs (74, 76). Eye movements frequently take place before a hand movement and may be spatially fixated on the object of interest until the end of reaching to improve accuracy (77–79). Decoupling of eye and hand movements requires reach and saccade goal separation (80–83). SPOC is more active in reaches toward peripheral (non-foveal) targets independent of gaze signals, while mIPS is more active in reach toward foveated targets with spatial congruence between gaze and reach goal (74).

In cortical reach-dominant regions, the anterior precuneus (aPCu) area, expanding into the medial IPS, is equally active in visual and non-visual reaching. Medial, anterior intraparietal and superior parietal cortices are also activated in both visual and non-visual reaching; areas located in the anterior distribution are more active during hand movements in comparison to those in the posterior distribution, which are more active during combined eye and hand movements. Another area, at the superior end of the parieto-occipital sulcus (sPOS), is more active during visual reaching. Taken together, aPCu may be a sensorimotor area with a prominent proprioceptive sensory input and sPOS, a visuomotor area that receives visual feedback during reaching (84).

In addition to these cortical contributions, the cerebellum plays a critical role in the timing and control aspects of manual dexterity, particularly multijoint movements, through both reciprocal connections with frontal motor areas, and through connections to the descending motor pathway through the red nucleus (85). The cerebellum receives inputs from a cortical network composed of motor, somatosensory, and posterior parietal areas via the pons. These inputs allow the cerebellum to compare the desired consequences of a movement (e.g., touching an elevator button), with the future progression of the hand through space as predicted from current motor commands. The cerebellum is often said to act as a “forward modeler” of the arm/hand for this reason (it can predict the consequences of the descending motor commands sent to the arm) (86, 87). The cerebellum is then able to modulate the ongoing stream of motor commands to correct anticipated errors, either through connections to SMA, or via a more direct modulation of the descending motor pathway via the red nucleus (86). Cerebellar damage results in motor incoordination, and a loss of the typical smoothness of manual motor trajectories through space (85, 88).

This highly interconnected reach network is further complicated by additional interconnections with the basal ganglia, the set of subcortical structures including the striatum (caudate nucleus and putamen), globus pallidus, subthalamic nucleus, and substantia nigra. Inputs to this functional grouping of nuclei from reach-related cortical areas are received by the striatum and processed by the remaining basal ganglia before being returned to the cortex (SMA) via the thalamus. The basal ganglia have a complex modulatory role in the reach motor network that appears to involve the choice of which movement to make, from among the possible alternatives, as well as the related function of assigning values to different possible movements (e.g., based on which are expected to be most rewarding) (89, 90).

## Sensorimotor Control: Ocular (Eye) and Manual (Hand)

### Overview

In humans, sensorimotor control strategies are essential for skilled somatic behavior; object manipulation performance aids in characterizing the interactions between the body and the article of interest (91). Before initiating a manual motor movement, the eyes very often fixate on the preferred object (92); however, a more invariant feature is that the eyes will spatially direct gaze on the target prior to the arrival of the hand (93), typically near the peak acceleration of the reach (94–96). The ocular motor system enables the needed visual information to direct the hand and successfully accomplish the task requirements; this is performed so fixations are “just in time,” providing information at the moment the additional foveal-based fine detail would be required for the task at hand (97). Change blindness and short-term memory limitations, features of normal visual function, support the notion that information acquired during prior fixations factors minimally into computation (98–100). The information that is integrated across the fixations when a visual scene, for example, is largely semantic in nature, i.e., the memory of an object’s identity but not specific features or the memory of a global scene but not particular details (101, 102). Therefore, eye movements are closely coupled to motor action in both time and space (103).

Sensorimotor coupling involves the fusion of visual perception and somatic motor control for action planning and behavioral execution; in fact, vision may best be understood through the “lens” of action production (104, 105). The line of sight is often directed at items of interest in an environment, upon which manual interactions may subsequently be focused. Based on the final goal of an intended manual interaction, grasping choices will be affected; this not only has relevance for motor control and planning requisite to finger position but also for eye fixation position, as gaze is paramount to precise manual action before execution (71). Eye fixations suggest a multitiered manual motor planning hierarchy. At the first level, it is determined where to grasp the object of interest, given the current descriptive content and the orientation of the object. If needed, at the second level the grasp is altered based on the type of secondary task to be accomplished with the grasped object, e.g., tool movement from location A to B. If needed, at the third level the movement plan incorporates a joint action component reflecting, e.g., the final resting place for the tool, handing it to a second person or placing it in a convenient location. Changes in the second and third levels of motor planning alter eye movement patterns and suggest a bidirectional sensorimotor coupling of eye to hand in coordinated activities (71).

The brain putatively plans visually guided action in the PPC, as suggested by neurophysiological studies in non-human primates, in imaging studies in healthy humans, and in human patients with cerebral injuries (74, 106–108). In non-human primate studies, electrophysiological results have revealed effector-specific regions in the PPC, with the parietal reach region relating to arm movements and the LIP area relating to saccadic activity. Given the relationship to effector preference but not dominance in these PPC subregions, functional imaging studies have sought to determine similar degrees of effector selectivity in human PPC, including area V7 and IPS areas 1 and 2 (IPS1 and IPS2) (109). Results indicate a limited degree of effector selectivity in the cortex and that transitions from the specificity surrounding one effector to another are gradual through the cerebral hierarchy in association with the frontal, parietal, and occipital cortices (109). In the visual cortex, there is a general preference noted for saccades, the PPC subregion, V7, has been specifically noted to activate relative to these fast eye movements. In the parietal cortex, IPS1 reflects a balance of saccade and reach activity, while IPS2 appears to be biased somewhat toward representing reach planning. In the frontal cortex, while regions near the central sulcus are more active for reach, FEF displays no effector preference (109), which may indirectly indicate a form of balance between eye and hand (Figure 2).

The PPC is of central importance given its strong feedforward connections to premotor and primary motor cortex (110). It has been suggested that the cytoarchitecture of networks between frontal and parietal cortices and their associative connections is ideal for integrating visual and somatic information (111). In fact, connections between the parietal and the dorsolateral (e.g., PMC) and medial (e.g., SMA) frontal motor areas may link vital neural information that assists in determining the visually deciphered target location and the somatic hand configuration required for execution (112). Expanding the integration network, the parieto-occipital junction shows activation when hand-motor goals are directed by a combination of gaze-oriented and proprioceptive body cues, suggesting some level of segregation within the reach-related regions of the PPC, while purely gaze-centered motor goals demonstrate activation in the anterior cuneus (113).

In visually guided reaching, studies in the macaque have shown that the ventral aspect of the parieto-occipital sulcus may act as a potential early node of the distributed eye–hand network, serving as a possible source of visual- and eye-position signals to parietal and frontal areas; this process has been described as re-entrant signaling, reciprocal associative connections leading to the interaction of eye and hand motor commands (110, 114). The ventral bank of the parieto-occipital sulcus, areas V6 and V6A, operates as an integrator of visual and somatic spatial information (115). There might be overlap between these two areas and the “parieto-occipital area” (PO) (116), but recent studies comparing the connections emphasize that V6Av (the ventral subregion of area V6A) is cytoarchitectonically and functionally distinct from the adjoining areas (V6 and V6Ad, the dorsal subregion) (117). More specifically, V6Av may serve as an integrator of visual and somatic/motor inputs (118). PPC is not only considered the sensorimotor interface for the planning and control of visually guided movements, but also conveys initial sensory-to-motor signals and online updates for the integration of sensory information from prior and current manual motor movement (119). The spatial position of the target is compared to the current spatial position of the hand which is thought to be represented in an eye-centered reference frame, mapping directly into motor error signals in a hand-centered reference frame; the superior parietal lobule (SPL) in the PPC is the primary location where these transformations are thought to occur with activation patterns mapped along a ventral–dorsal axis (119).

### Coordinate Mapping Based on Visual Cues

Visual cues that translate into retinotopically coded information must be converted into meaningful output for effector-specific, goal-directed activity. The PPC may direct and plan movement by establishing a head/body-centered coordinate system, through both visual input and motor/proprioceptive cues, or, in contrast, utilize an eye-centered coordinate system (120, 121). An eye-centered frame of reference proves useful when considering the optimal dual-effector coordination, as eye movements are coded in eye-centered coordinates: extending this into PPC would ostensibly be strategic (122). In addition, the eye-centered reference frame used in PPC would help in accounting for online obstacles during visuomotor action and during error correction (123, 124). Evidence from macaque supports the concept of PPC operating under a common reference frame, where sensory targets are computationally processed for transformation from head-, body-, eye-, and limb-based coordinates into one eye-centered representation; this simplifies inter-effector motor planning (122).

The brain maintains a dynamic map of memory-based, geometric space in a gaze-centered coordinate system (125). On a cellular level, in the primate parietal cortex, the receptive fields of neurons have been shown to shift transiently in anticipation of an eye movement, predicting the sensory consequences of the intended eye movement (126). Given natural delays in sensory feedback and the anticipatory nature of this physiologic phenomenon, the mechanism is likely a forward model similar to what has been described for arm movements (87, 127, 128), which would combine sensory feedback with the predicted consequences of motor commands to facilitate online feedback control; additionally, this may impact the process by which the brain monitors and stores memories of previous movement execution and performance (125, 129, 130).

## Impairment of the Visuomotor System

### Pathology and Clinical Disease

Pathology and clinical disease provides neuroanatomical and neurophysiological “knockouts” that can be diagnosed and characterized behaviorally, shedding light on cerebral function. Connecting empirical data on clinical deficits with neuroimaging and anatomical correlates yields greater understanding behind the nature of specific visuomotor pathologies and more significantly on relevant connections, associations, pathways, and networks. Optic ataxia (OA), as a clinical entity, is an archetype; patients demonstrate difficulty in executing visually guided reaching without additional sensory cues, accompanied by deficits in prehension and hand orientation. As opposed to Balint’s syndrome or OA plus ocular apraxia and simultagnosia, an isolated optic ataxia often manifests with intact ocular motor function, full visual fields, normal depth perception, complete motor ability, and cerebellar function and no known cause of reaching ataxia. These clinical signs and disease patterns are attributed to lesions in the PPC or, more specifically, neurovascular injuries in the superior and inferior parietal lobule (SPL and IPL, respectively), around the IPS (131–134).

Optic ataxia, again defined as the inability to properly reach or grasp objects under visual control, particularly under peripheral vision, is associated neuroanatomically with dysfunction at the border of the SPL, near the IPS, but superior to the IPL, and behaviorally with poor motor performance when faced with moving targets that pose immediate motor programming challenges (135, 136). More precisely, the SPL receives afferent signals from the extrastriate areas of the occipital lobe and has reciprocal connections to and from the premotor and primary motor cortices of the frontal lobe, serving as a multisensory integration hub planning motor commands (137, 138). Optic ataxia has been interpreted as a combinatorial dysfunction in the ability of parietal neurons to integrate retinal, eye, and hand signals utilized for EHC (134). The neural mechanisms of hand movement corrections given rapid target changes shed light on the functional abilities of the eye and hand to maintain coupling and assist in further understanding the pathology of optic ataxia, highlighting clinical deficits that manifest as an inability to quickly adjust in-flight hand movement trajectories aimed at moving objects (132).

### Sensorimotor Impairment

Cerebrovascular accident leads to sensorimotor impairments that result in a myriad of deficits in visually guided reaching and pointing movements, impairments that are noted in both the contralateral and ipsilateral hands (2, 139–145). The focus post-injury has been to examine the hand objectively during visually guided action without objective eye movement assessment, leaving one to question the abnormalities that may exist between effectors. In fact, the ocular motor system, when objectively assessed, has been shown to be a powerful tool in clinical neuroscience, serving as a marker of cerebral function (146–148). Recently, eye movements have been shown in stroke investigations to be a sensitive biomarker for cognitive and motor recovery (149, 150). Additionally, poststroke patients display unique pathophysiologic phenotypes that may include tactile deficits (151–153), proprioceptive losses (154, 155), hemiparesis and related motor synergies (156–158), and spasticity (159–161), which would suggest that these new sensory and motor “states” postinjury create new relationships between receptor and effector, requiring the need for re-integration (162–164).

In fact, poor visuomotor performance (EHC) has been associated with poorer accuracy and longer movement times in visually guided action poststroke, and these deficits have correlated significantly with impairments at the sensory and motor level; more specifically, poor chronometric and spatial performance in the more affected limbs of stroke subjects have correlated with tactile insensitivity, handgrip strength deficits and more severe motor impairment scores, as assessed by the Fugl-Meyer (2). It is well known that reaching depends on inputs from both vision and proprioception; tactile sensation is a component of proprioception, particularly when proprioceptive inputs may be impaired (165, 166). Evidence of this sensori-motor coupling in control physiology during multi-joint action tasks is well documented (167, 168). Optimality in functionally oriented somatic movements of the upper extremity is demonstrated through hand paths that are straight, smooth, and with bell-shaped velocity profiles that scale with distance, implying advance planning (169, 170). These control markers set comparative baselines for investigations into impairment and not surprisingly suggest impairments in motor control programming at the planning level and at the sensori-motor interface level (162, 171, 172).

Following stroke, sensorimotor uncoupling is a byproduct of new relationships between impaired sensory input and poor motor output (163). As these new relationships are learned, the execution of limb movements is altered, above and beyond what would be expected from the individual deficits themselves: take for example a velocity curve that has an earlier peak and a prolonged deceleration phase, allowing greater opportunity for feedback mechanisms to improve endpoint accuracy (141, 163, 173). An intriguing experimental paradigm is the double-step saccade task (174), in that goal-directed action can be tested while a spatial target is displaced between two locations during the primary saccade, a period in which there is no visual perception. This paradigm can be deployed as a part of a visually guided reach to point task and will decrease the performance of the arm movement without mechanical perturbation or cognitive understanding of the manipulation. It has been suggested that during visually guided rapid arm-movement control, in which saccadic double-stepped targets are implemented, that spatial corrections of the hand are driven by ocular motor corrections following spatial target shifting (175).

Vision is essential to the sensori-motor integration required for visuomotor action. Gaze position is a consequence of ocular motor control and supports hand movement planning. These spatial locations or fixations often mark key positions for fingertip placement and are a byproduct of the functional requirements of the task at hand (91, 176). Furthermore, vision-based hand feedback is vital to motor adjustments during online control, as saccadic behavior updates spatial understanding and improves goal localization (177); in fact, it has been suggested that there is parallel processing between effectors (77, 178). This could be particularly problematic in patients with ABI with eye movement deficits (179–185), in addition to somatic motor deficits (e.g., hemiparesis). While manual motor deficits are typically evident on clinical examination, ocular motor deficits frequently require objective recording techniques (186–194) for identification and prognostication (181, 183, 195). Nevertheless, even if eye movements are found to be sound post-ABI, clinically and subclinically, following objective recordings, an impaired limb with poor functional performance may lead to maladaptive ocular motor behavior to compensate for lost task ability.

An eye-hand dyssynergia, or a lack of coordination between effectors, may operate in suboptimal modes to re-establish premorbid skill level, impeding recovery. This sensorimotor impairment may by multifactorial and compromised secondary to not only ocular motor deficits but also visuospatial and visuoperceptual abnormalities (196–200), in addition to balance deficits; in fact, decreases in balance have been noted during EHC tasks with stroke patients (142). This may all be of significant interest given the increased sensitivity during poststroke periods to sensory reweighting (201).

### Deficits of Predictive Control

Our visual world is ever changing and prediction is a necessary part of object manipulation and consequently an important aspect of eye–hand control. Catching a ball or grasping a pen being handed to you requires anticipating the motion and direction of the object, and planning a motor response that will intersect successfully with the predicted trajectory. If the afferent end of visual processing or perception was simply used to generate spatial cues for EHC, our hand would consistently miss the spatial target; rather, an integrated construct replete with anticipation and prediction is pivotal to successful outcomes, which translate into functional performance (202). Superior skill in sports demonstrates finely tuned ocular motor control that drives complex somatic motor control (203–207). For example, soccer goalkeepers at the expert level demonstrate more accurate soccer ball prediction during anticipation tasks, as compared to novice level players; differences also include efficient and more effective strategies during visual search, which consist in part of longer fixations that are less frequent and directed at disparate regions of interest (208, 209).

In ABI, including injuries secondary to neurovascular and neurotraumatic insults significant predictive control deficits have been demonstrated during dynamic EHC tasks in the absence of deficits during static visuomotor tasks, highlighting difficulties in rapidly processing sensory information rather than motor execution errors. Delaying or inefficiently managing sensory information may not only lead to problems with target anticipation during dynamic tasks (feedforward impairment), but also the use of sensory feedback for error correction (1, 210). In fact, studies have demonstrated ocular motor deficits in predictive control within ABI for moving targets with and without intermittent stimulus blanking, and these impairments have been correlated with cognitive performance (211–213). Moreover, increasing cognitive load during predictive ocular motor tasks degrades performance in ABI and may suggest an “overload” to the impaired neural network (214).

This opens several broader questions, as patients with ABI who suffer from impaired eye movements, or even decreases in exploratory eye movements, may have perceptual limitations that hampers the understanding of scenes and spatial relationships between objects. This, in combination with loss of sensory feedback systems typically in place during action production, may increase the cognitive complexity of the task at hand (3). This may be more problematic in tasks for which EHC needs to flexibly convert from coupled function to uncoupled or decoupled function. For example, consider reaching for your cell phone while reading a newspaper, thus executing a somatic motor movement toward one spatial target while simultaneously executing saccades during the reading task elsewhere (74). Even asymptomatic post-ABI patients have shown difficulty in visually guided action when there is a level of dissociation between the visual information used to guide the required motoric action, decoupling the eye and the hand and perhaps increasing the task complexity. Similarly, multidomain tasks that encompass cognitive and motoric skill are effective at “pushing” the brain during functionally relevant performance; these constructs must be viewed on a spectrum. A cognitive “load” in such dual tasks can be experimentally manipulated and made more or less challenging for more effective screening; at the mild end, this may be accomplished by increasing the cognitive difficulty (e.g., visually guided pointing coupled with a serial sevens countdown), or decreased for those on the severe end by decreasing the cognitive difficulty (e.g., adding an easily predictable element to a spatial sequence of visually guided pointing) (215).

These predictive control deficits are provocative when framed in neurovascular and neurotraumatic conditions, particularly when visually guided action is uncoupled and spatial targets are dynamic. However, in the setting of neurodegeneration, whether one considers vascular dementia following repeated multistepped strokes or chronic traumatic encephalopathy following repeated traumatic brain injuries, these constructs are even more compelling, given the cognitive impairments that may be superimposed on ocular motor and/or visual deficits (216–219).

### Disorders in Visuomotor Planning

Predictive control is a central element of visuomotor planning; this is particularly relevant during dynamic motor tasks with spatial targets that are in motion and that require anticipation for successful interaction (202). However, at a more basic level, if one considers motor programming or feedforward control during tasks without dynamically moving targets, the planning of hand movements during reach is impaired after stroke or post-ABI (171, 172, 220, 221). Impaired planning results in an inability to program sequences of motor action in space and time (139, 222–225). As the environment undergoes incessant change, our body must adapt, a fundamental element to spatially accurate motoric action. During adaptation, previously observed errors in one’s own performance inform the correction of future motor plans. It has been suggested that sensory prediction errors are a primary input for motor programming revisions, during which planning is adjusted following a comparison between motor output and the predicted sensory outcomes of the original plan (226).

While planning is contingent on sensory information, e.g., vision and proprioception, laterality may also play a significant role. It has been even suggested that hemispheric specialization is paramount, producing dissociable differences in poststroke motor control. The left hemisphere is theorized to be motor-planning dominant for feedforward control while the right hemisphere is theorized to be feedback dominant for error correction during position control. Following this construct, a limb stabilized on a visual target may leverage right hemisphere resources, while a limb attempting to catch a moving ball may leverage left. In concert, optimizing ongoing action is undoubtedly the integration of feedforward and feedback control, and ABI has revealed deficits in initial trajectory profiles in left-brain injury and deficits in spatial accuracy in right-brain damage (227–230). Thorough assessment and targeted treatment of planning deficits may lead to improved motor relearning and functional recovery in ABI (221).

### Clinical Implications and Outcome Measures

Acute and chronic disease processes that lead to cerebral injury are often challenging from a diagnostic and therapeutic standpoint; this is particularly true with neurodegenerative disorders secondary to their often diffuse and indolent nature, constraining our ability to isolate specific structural abnormalities with associations to functional limitations (231). To improve our understanding of neurophysiology and enhance our understanding of the clinical implications, experiments have historically concentrated on focal lesion-based approaches. These lesion-based models and associated biomarkers can be combined synergistically with the goal of detecting and characterizing the preclinical evolution of the neurobiological events that precede the cognitive impairments associated with neurodegeneration (232–235).

Objective eye movement recordings, when approached with methodological rigor, have already proven valuable as a research tool within ABI (236–238). In fact, ocular motor recordings have been used for their screening utility in a diagnostic capacity (211–214). As a response, rapid, vision-based performance measures that depend on time taken and errors made during visually presented number reading or object naming have been developed and extensively studied in the setting of ABI (239–244). More broadly, eye movements and visuomotor skill of the upper limb have been sensitive markers of cerebral injury (245). Taken further, eye and arm function following acute ABI has demonstrated good predictive capacity for outcomes in the subacute and chronic stages following injury with superior performance when compared to health status on self-report or based on neuropsychologic assessment (1, 246, 247). These prognostic capabilities have also enabled the identification of individuals who are poor responders or those who may require more aggressive intervention (248, 249). Ocular motor performance has even been demonstrated to be a biomarker of cognitive recovery beyond the times at which apparent full recovery had been deemed, as assessed by conventional metrics (150). While the literature is more extensive for neurovascular and neurotraumatic etiologies, the evidence base does extend into neurodegeneration (250, 251).

Given this framework, it is not difficult to see that there are extensive opportunities for translational ocular motor investigations that extend beyond the research setting and into the clinic. These opportunities are multiplied when ocular motor investigations are juxtaposed with manual motor investigations in ABI. While the clinical implications are significant, the literature has yet to see objective ocular motor and somatic motor control recordings enter the setting of ABI for unconstrained, coordinated eye and hand movements and frequently the motor output that is quantified during visually guided action is simply somatic in nature (Table 2). Though examples certainly exist where these two effectors have been objectively recorded, the movements have been constrained to one or two spatial dimensions, limiting the ecological validity; such constrained movements may require altered programmatic control between effectors, as a limb restricted to execute somatic motor output in an unnatural mode may have problematic effects on the ocular motor output, restricting comparisons. In the present narrative review, there was only one study that simultaneously recorded ocular and manual motor activity; the remaining manuscripts quantified movements of a single effector system (Table 2).

In fact, objective EHC tasks have already been designed for neurodegenerative disease processes, incorporating simultaneous ocular and manual motor recordings (252, 253). These investigations focused on integrated eye and hand assessments have yielded promising results and used simple tasks during which subjects are merely asked to perform a “look and reach,” revealing quantifiable deficits in visually guided action (254). Additionally, and perhaps more promising, are tasks that have combined more cognitively demanding elements, e.g., antisaccades and antitapping, as part of an effort to increase the diagnostic power of the measures (252, 253, 255). While it has been suggested that objectified visuomotor tasks and related deficits may assist in diagnosing prodromal neurodegenerative disease entities and monitoring their progression, similar tasks that further increase complexity with distractors and/or feedback perturbations may assist in preclinical detection.

Currently, a central focus of rehabilitative interventions for cerebral injury is to restore motor ability and increase function. However, the return of motor ability often does not ensure ecologically valid, meaningful gains in function (222, 256). A clearer characterization of ocular motor control and its relationship to manual motor control will improve our understanding of EHC in a functional context. The quantitative relationships and motor outputs from both effectors are likely to yield metrics that can be correlated and compared to existing assessments and outcome measures utilized in current care models. Positive relationships will yield significant opportunities on the diagnostic, prognostic and therapeutic fronts, driving toward the development of algorithmic approaches with tailored, patient-specific management plans.

## Conclusion

During goal-directed movement, first-rate function often requires that visual perception, under precise ocular motor control, be translated optimally into somatic action. Leveraging focal lesion-based models and associated eye–hand biomarkers is a robust approach toward significantly improving our understanding of acute and chronic neurological disease processes. In recent years, a number of studies have focused on EHC in ABI. The present review describes a series of studies that directly or indirectly highlight EHC in ABI and the neuroanatomic, computational, and broader clinical implications. While there is ample evidence to suggest that coupling is essential to EHC and that it is a sensitive biomarker for cerebral injury, visually guided action in the experimental setting has typically been limited to quantifying one effector or two effectors in a limited or constrained fashion. As such, it is recommended that future studies addressing related behavior should concurrently objectify ocular and manual motor control in unconstrained and natural modes. These studies, while technically more challenging, are likely to further characterize coupling and potentially yield high-impact results along the care spectrum from diagnosis to neurorehabilitative treatment in the setting of neurovascular, neurotraumatic, and neurodegenerative pathology.

## Author Contributions

Conception and design of the review: J-RR and EW. Substantial manuscript drafting: J-RR, MH, JF, WM, EA, PR, JR, ML, and TH.

## Conflict of Interest Statement

The authors declare that the research was conducted in the absence of any commercial or financial relationships that could be construed as a potential conflict of interest.
